# Supplementary material for: Cortical signatures of precision grip force control in children, adolescents, and adults
Source: eLife. 2021 Jun 14;10:e61018. doi: 10.7554/eLife.61018 (PMC8216716; doi:10.7554/eLife.61018)
Supplement: Supplementary file 1. [file elife-61018-supp1.docx]

**Supplementary file 1**

**Supplementary Table S1: Table representing the pipeline used in EEGLAB (v. 14.1.1) interfaced in Matlab R2017b**

| Process | | | Function | | Settings/options | |
| --- | --- | --- | --- | --- | --- | --- |
| Import data | | | pop_biosig | |  | |
| Filter from 0.5 to 48Hz with FIR filter | | | pop_eegfiltnew | | Automatic filter order by EEGLAB | |
| Add channel locations | | | pop_chanedit | |  | |
| Visual inspection of noisy channels | | | pop_eegplot | |  | |
| Remove noisy channels | | | pop_select | |  | |
| Periods with high-amplitude deflections removed | | | pop_select | |  | |
| Average reference | | | pop_reref | |  | |
| Downsample from 2048Hz to 256Hz | | | pop_resample | |  | |
| ICA decomposition | | | pop_runica | | *runica* algorithm | |
| Plot and remove components representing eye-blinks and saccades | | | pop_plot; pop_subcomp | | Identified based on (Chaumon et al., 2015) | |
| Interpolate removed channels (if any). | | | pop_interp | | *spherical* algorithm | |
|  |  |  |  |  |  |  |
