# Supplementary material for: Cortical signatures of precision grip force control in children, adolescents, and adults
Source: eLife. 2021 Jun 14;10:e61018. doi: 10.7554/eLife.61018 (PMC8216716; doi:10.7554/eLife.61018)
Supplement: Supplementary file 2. [file elife-61018-supp2.docx]

**Supplementary file 2**

**Supplementary Table S2: Table representing steps applied during conversion of files to SPM data files in SPM12 (v7487) interfaced in Matlab R2017b**

| Process | Function | Settings/options |
| --- | --- | --- |
| Convert to spm data file | Batch tool ('Conversion') | Default settings |
| Epoch data | Batch tool ('Epoching') | Arbritrary trials'. Trial length set to 1000ms |
| Convert sensors to MNI space | Batch tool ('Prepare') | Assign default EEG sensors |
