# Supplementary material for: Cortical signatures of precision grip force control in children, adolescents, and adults
Source: eLife. 2021 Jun 14;10:e61018. doi: 10.7554/eLife.61018 (PMC8216716; doi:10.7554/eLife.61018)
Supplement: Supplementary file 3. [file elife-61018-supp3.docx]

**Supplementary file 3**

**Supplementary Table S3: Table representing the pipeline used for estimating single-subject DCMs in SPM12 (v7487)**

| Process | Function | Settings/options |
| --- | --- | --- |
| Specify 'template' DCM | SPM GUI ('Dynamic Causal Modelling') | CSD' and 'LFP'; Time window = 1000 ms; detrend = 1; subsample = 1; modes = 7; electromagnetic model = 'IMG'; Frequency band = 4-48Hz |
| Set hyperprior for noise precision (hE) | spm_dcm_csd | line 127; hE=18 |
| Set prior of neural innovations to flat spectrum | spm_dcm_csd | line 127; pE.a(2,:)=-32 |
| Set up for parallel processing | spm_dcm_fit | line 24 changed from 'FALSE' to 'TRUE' |
| Estimation of single-subject DCMs | Batch tool | Collate to one GCM. |
