# Supplementary material for: Cortical signatures of precision grip force control in children, adolescents, and adults
Source: eLife. 2021 Jun 14;10:e61018. doi: 10.7554/eLife.61018 (PMC8216716; doi:10.7554/eLife.61018)
Supplement: Supplementary file 4. — *The parametric empirical Bayes analysis was run three times, with the design matrix reordered so that the main regressor of interest was listed just after the commonalities (column 2) as per https://en.wikibooks.org/wiki/SPM/Parametric_Empirical_Bayes_(PEB)#Search_over_nested_PEB_models. [file elife-61018-supp4.docx]

**Supplementary file 4**

**Supplementary Table S4: Represents the pipeline used for the group-level (2^nd^ level) analyses of DCMs in SPM12 (v7487)**

| Process | Function | Settings/options |
| --- | --- | --- |
| Specify PEB | SPM GUI ('Dynamic Causal Modelling') | The GCM containing 88 subject-specific DCMs were entered. A 88x4 design matrix was entered with the following columns*: (1) Commonalities (all 1s), (2) Dummy coded age group (mean-centered), (3) Precision score (mean-centered), (4) Interactions between age-group and performance (product of the two; mean centered). We set Fields = {'A'} to model extrinsic connections. We used the 'default' settings for the remaining settings. |
| Model comparisons: Search nested PEB models (greedy search scheme). | spm_dcm_peb_bmc | Default options used |
| Review results | spm_dcm_peb_review |  |
| Threshold results | spm_dcm_peb_review | > 95% posterior probability using the 'Free Energy' option |
| * the PEB analysis was run three times, with the design-matrix re-ordered so that the main regressor of interest was listed just after the commonalities (column 2) as per <https://en.wikibooks.org/wiki/SPM/Parametric_Empirical_Bayes_(PEB)#Search_over_nested_PEB_models> | | |
