# Supplementary material for: Cortical signatures of precision grip force control in children, adolescents, and adults
Source: eLife. 2021 Jun 14;10:e61018. doi: 10.7554/eLife.61018 (PMC8216716; doi:10.7554/eLife.61018)
Supplement: Transparent reporting form [file elife-61018-transrepform.docx]

***eLife’s* transparent reporting form**

We encourage authors to provide detailed information *within their submission* to facilitate the interpretation and replication of experiments. Authors can upload supporting documentation to indicate the use of appropriate reporting guidelines for health-related research (see [EQUATOR Network](http://www.equator-network.org/%20)), life science research (see the [BioSharing Information Resource](https://biosharing.org/)), or the [ARRIVE guidelines](http://www.plosbiology.org/article/info:doi/10.1371/journal.pbio.1000412) for reporting work involving animal research. Where applicable, authors should refer to any relevant reporting standards documents in this form.

If you have any questions, please consult our Journal Policies and/or contact us: [editorial@elifesciences.org](mailto:editorial@elifesciences.org).

**Sample-size estimation**

- You should state whether an appropriate sample size was computed when the study was being designed
- You should state the statistical method of sample size computation and any required assumptions
- If no explicit power analysis was used, you should describe how you decided what sample (replicate) size (number) to use

Please outline where this information can be found within the submission (e.g., sections or figure legends), or explain why this information doesn’t apply to your submission:

- Given that, to the best of our knowledge, no previously published study has investigated age-related differences in brain connectivity in typically developing children, adolescents and adults we could not perform any well-informed power analysis prior to undertaking the experiment. We therefore aimed at recruiting at least 20 individuals in each group based on a pragmatic trade-off between amount of data (and statistical power) and the time taken to perform the experiments.

**Replicates**

- You should report how often each experiment was performed
- You should include a definition of biological versus technical replication
- The data obtained should be provided and sufficient information should be provided to indicate the number of independent biological and/or technical replicates
- If you encountered any outliers, you should describe how these were handled
- Criteria for exclusion/inclusion of data should be clearly stated
- High-throughput sequence data should be uploaded before submission, with a private link for reviewers provided (these are available from both GEO and ArrayExpress)

Please outline where this information can be found within the submission (e.g., sections or figure legends), or explain why this information doesn’t apply to your submission:

- Criteria for inclusion and exclusion has been listed in the ‘Methods and materials’ section under the subheading ‘Participants’. Reasons for exclusion of obtained datasets are outlined in the results section.

**Statistical reporting**

- Statistical analysis methods should be described and justified
- Raw data should be presented in figures whenever informative to do so (typically when N per group is less than 10)
- For each experiment, you should identify the statistical tests used, exact values of N, definitions of center, methods of multiple test correction, and dispersion and precision measures (e.g., mean, median, SD, SEM, confidence intervals; and, for the major substantive results, a measure of effect size (e.g., Pearson's r, Cohen's d)
- Report exact p-values wherever possible alongside the summary statistics and 95% confidence intervals. These should be reported for all key questions and not only when the p-value is less than 0.05.

Please outline where this information can be found within the submission (e.g., sections or figure legends), or explain why this information doesn’t apply to your submission:

- The methods used for the statistical analyses is accounted for in the Methods and Materials section under the subheadings ‘Precision grip force task’ for behavioral data and ‘Group level analysis using Parametric Empirical Bayes (PEB)’ for electrophysiological data.

- Individual ‘raw’ data is included in figures presenting key outcomes presented throughout the manuscript where appropriate.

- Information on statistical test used including corrections for multiple comparisons are explicitly stated at the beginning of each subsection throughout the Results section. The exact number of participants (N) is presented in Table 1. Data from all these participants are used throughout the manuscript. Precision and dispersion measures are defined in figure legends and table descriptions where appropriate.

- Exact p-values are reported where appropriate (e.g. Results section where task performance differences are presented). This is the case for all comparisons using frequentist approach. Statistical inference in the DCM analysis is Bayesian, and therefore exact posterior probabilities are presented (Results section).

(For large datasets, or papers with a very large number of statistical tests, you may upload a single table file with tests, Ns, etc., with reference to sections in the manuscript.)

**Group allocation**

- Indicate how samples were allocated into experimental groups (in the case of clinical studies, please specify allocation to treatment method); if randomization was used, please also state if restricted randomization was applied
- Indicate if masking was used during group allocation, data collection and/or data analysis

Please outline where this information can be found within the submission (e.g., sections or figure legends), or explain why this information doesn’t apply to your submission:

- Individuals were recruited to the experiment based on their biological age.
- Masking were not used, as this was deemed incompatible with the overall aim of studying age-related differences, as blinding of both participants and experimenters is not feasible due to characteristic age differences.

**Additional data files (“source data”)**

- We encourage you to upload relevant additional data files, such as numerical data that are represented as a graph in a figure, or as a summary table
- Where provided, these should be in the most useful format, and they can be uploaded as “Source data” files linked to a main figure or table
- Include model definition files including the full list of parameters used
- Include code used for data analysis (e.g., R, MatLab)
- Avoid stating that data files are “available upon request”

Please indicate the figures or tables for which source data files have been provided:

- Numerical data used in Table 1; Figure 1D; Figure 4; Figure 5 and Figure 6 will be uploaded as .csv files.
- The R code used for the data analysis and Matlab code for preprocessing of EEG data will be uploaded.
